# Sensitive Carbon Fiber Microelectrode for the Quantification of Diuron in Quality Control of a Commercialized Formulation

**DOI:** 10.1155/2022/9994639

**Published:** 2022-03-11

**Authors:** Serge Mbokou Foukmeniok, Roland Fabrice Yibor Bako, Ousmane Ilboudo, Yssouf Karanga, Evangéline Njanja, Maxime Pontie, Issa Tapsoba, Ignas Tonle Kenfack, Rousseau Djouaka

**Affiliations:** ^1^Electrochemistry and Chemistry of Materials, Department of Chemistry, University of Dschang, P.O. Box 67 Dschang, Cameroon; ^2^Laboratoire de Chimie Analytique Environnementale et Bio-Organique (LCAEBiO), Département de Chimie, Université Joseph KI-ZERBO, 03 BP 7021 Ouagadougou 03, Ouagadougou, Burkina Faso; ^3^Angers University, Group Analysis and Processes (GA&P), Chemistry Department, Faculty of Sciences, 2 Bd A. de Lavoisier, 49045 Angers Cedex 01, France; ^4^International Institute of Tropical Agriculture, Cotonou 08 BP 0932, Benin; ^5^Ecole Normale Supérieure (ENS), 01 BP 1757 Annexe Ouagadougou, Secteur 29, Burkina Faso; ^6^Laboratoire de Chimie des Matériaux et de l'Environnement (LCME), Université Norbert ZONGO, Avce Maurice Yameogo, Koudougou BP 376, Burkina Faso

## Abstract

Nickel(II) tetrasulfonated phthalocyanine (p-NiTSPc)-modified carbon fiber microelectrode (CFME) was used for the first time to investigate the electrochemical quantification of diuron in an agrochemical formulation. The surface morphology and elementary analysis of unmodified carbon fiber microelectrode (CFME) and p-NiTSPc-CFME were performed using atomic force microscopy (AFM) and energy dispersive X-ray spectroscopy (EDX), respectively. Cyclic voltammetry (CV) was used to investigate the electrochemical behaviour of diuron, while square wave voltammetry (SWV) was used for quantitative analysis of diuron. Upon variation of some key analytical parameters, a calibration curve was plotted in the concentration range from 21.450 to 150.150 *μ*M, leading to a detection limit (DL) of 8.030 *μ*M mg/L (3.3*σ*/m) and a limit of quantification (LQ) of 20.647 *μ*M mg/L. The fabricated p-NiTSPc-CFME was successfully applied for quality control in a commercialized formulation of diuron. The standard additional method was used, and the recovery rate of diuron was found to be 98.4%.

## 1. Introduction

Pesticides are chemical substances used to control agricultural and public health pests. They can be classified according to their applications: to kill insects (insecticide), fungi (fungicide), weeds (herbicide), and rodents (rodenticide). Diuron (N-(3,4-dichlorophenyl)-N, N-dimethylurea) is a herbicide widely used in Africa by farmers for the control of weeds in crops such as soybeans, cotton, sugarcane, citrus fruit, wheat, and coffee [1–3]. It has been reported as a pesticide of great potential affecting human health [4]. Human exposure to diuron shows ecotoxic effects by acting as endocrine disruptors [5]. Diuron can also affects human health by causing irritation to the mucous membrane, skin, and eyes and in the bloodstream interacts with haemoglobin-forming methaemoglobin leading to loss of consciousness and abnormalities in the liver and spleen [6–8]. Moreover, the diuron overuse contaminates the environment and the food produced, posing a real public health problem. In Africa, diuron is marketed under various trade names, and most of the time, the values of standard inscribed on the packaging is not exactly that found in the container. This may affect plants, the environment, and farmers by decreasing agricultural yields, pollution of ecosystems, or exposing farmers to a diuron overdose [6]. Based on this, the development of sensitive and accurate electrochemical sensors for diuron determination is crucial for quality control of agrochemical formulations and for monitoring the environment. Numerous methods have been used for diuron detection including spectrometric [9], chromatographic [10], fluorimetric [11], capillary electrophoretic [12], and electrochemical [13] techniques. Electrochemical techniques based on chemically modified electrodes are much attractive due to their high sensitivity, rapid response, good stability, and reproducibility [14, 15]. Moreover, these techniques are recognized to be simple and inexpensive [15–17]. To the best of our knowledge and contrary to macrosized electrodes, microelectrodes offer numerous benefits, including the low ohmic drop, low capacitive current, and three-dimensional mass transportation that leads to a higher signal-to-noise ratio and a lower detection limit [18, 19]. In addition, because of their miniaturized size, microelectrodes are used with little amounts of analyte and could be useful for in vivo analyses [19–22]. The present study reports the development of nickel(II) tetrasulfonated phthalocyanine (p-NiTSPc)-modified carbon fiber microelectrode (CFME) for the electrochemical determination of diuron in aqueous solution. The developed sensor is successfully applied for quality control of diuron in a commercial formulation.

## 2. Materials and Methods

### 2.1. Reagents and Solutions

Carbon fibers (10 *μ*m diameter) used were purchased from Cytec Engineered Materials (West Paterson, NJ, USA). NiTSPc, Na_2_HPO_4_, and NaH_2_PO_4_ were provided by Sigma-Aldrich. The commercial formulation of diuron (ACTION 80 DF) was purchased from Louis Dreyfus Commodities agrochemical company, Burkina Faso. Diuron standard was provided by Sigma-Aldrich. A stock solution of diuron 0.5 g/L was obtained by dissolving 12.5 mg of the standard in 250 *μ*L of ethanol and diluting to 25 mL with distilled water. Taking into consideration the chemical structure of diuron, phosphate buffer solution (PBS; 0.1 M, pH 7.0) was chosen as a supporting electrolyte. In fact, diuron bears an amine functional group that could be interacting with the acidity of the supporting electrolyte by affecting its redox behaviour.

### 2.2. Commercial Agrochemical Formulation

A quality control analysis was achieved on a commercialized diuron formulation (ACTION 80 DF). To reach this aim, a stock solution of commercial diuron 1 g/L was prepared by dissolving 25 mg of the compound in 500 *μ*L of ethanol and diluting to 25 mL with distilled water. 200 *μ*L of the prepared solution was diluted to the mark with PBS in a 10 mL volumetric flask and transferred into the voltammetric cell. The resulting solution was firstly analysed by recording the corresponding square wave voltammogram on p-NiTSPc-CFME. Afterwards, the sample was spiked with known amounts of diuron standard, and the SWV of added concentrations of diuron was also recorded on p-NiTSPc-CFME. The unknown concentration of diuron in the commercial formulation was finally determined (standard addition method).

### 2.3. Apparatus

Electrochemical analyses were carried out on a DY2300 electrochemical analyser (Digi-IVY Instruments, USA) running with the DY2300 EN software and connected to a personal computer. A classical three-electrode cell configuration was employed, consisting of p-NiTSPc-CFME used as the working electrode, a saturated calomel electrode (SCE) as the reference electrode, and a platinum wire as the counter electrode. Atomic force microscopy (AFM) and energy dispersive X-ray spectroscopy (EDX), on a JSM-6301F apparatus from JEOL (SCIAM, University of Angers, France), were used for morphological studies and elementary analysis of unmodified and p-NiTSPc-modified carbon fiber microelectrodes, respectively. Modified and unmodified carbon fibers were immobilized on a sample holder using adhesive carbon tape. Images obtained were from secondary electrons of 3 keV, with magnifications between ×25 and ×20,000.

### 2.4. Preparation of Working Electrodes

The carbon fibers (length 6 mm) were firstly pretreated, and the procedure used was adapted from that described by Pontié et al. [17]. Briefly, the fiber was firstly treated in 0.1 M PBS at pH 7.0, then in a mixture of 0.5 M H_2_SO_4_/ethanol (1 : 1 v/v). Then, multisweep cyclic voltammetry was used in the potential range from 0 to 1.3 V/SCE, at a potential scan rate of 100 mV/s. A sequence of ten and five cyclic scans was used for the first and second pretreatment, respectively. The pretreated CFME was immersed afterwards into 0.1 M NaOH solution containing 2 mM NiTSPc, and the electropolymerization of the monomer was performed by 50 consecutive cyclic voltammetry scans over a suitable potential range of 0 to 1.3 V/SCE (scan rate 100 mV/s) [23].

## 3. Results and Discussion

### 3.1. Presentation and Characterization of Working Electrodes

**Figure 1:**
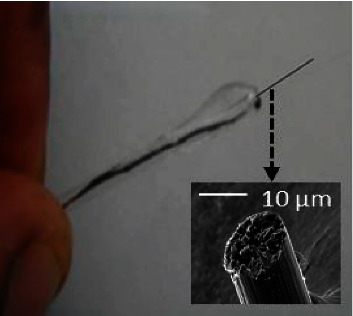
Image presenting the unmodified carbon fiber microelectrode. Inset shows the SEM image of the carbon fiber.

Figure 1 presents the image of the fabricated CFME, and the inset shows the SEM image of the unmodified carbon fiber. The CFME was made by connecting the fragment of a carbon fiber to a copper wire making use of a mixture of glue. The whole was carefully immobilized into a glass tube using the same mixture of glue. The copper wire serves to establish an electrical contact between the carbon fiber and the rest of the circuit.

**Figure 2:**
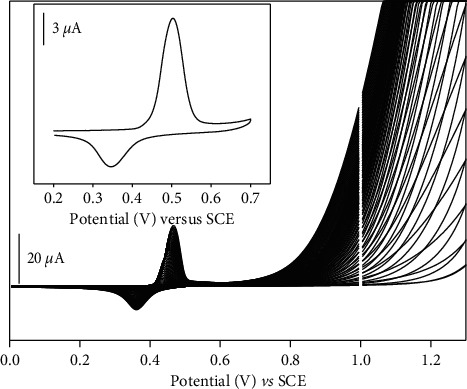
Electrodeposition of the p-NiTSPc film on the carbon fiber surface. Inset shows a cyclic voltammogram recorded on p-NiTSPc-CFME in 0.1 mol/L NaOH, after the electrodeposition of the p-NiTSPc film, with a scan rate of 0.1 V/s.

Once the CFME was made, the fiber was modified by electrodeposition of a poly-NiTSPc film on its surface. The aim of this modification was to enhance the CFME properties. Considering the fact that poly-NiTSPc bears different functional groups and negative charges [17], it is expected that a great affinity should be established between the electrode surface and the dissolved diuron. The electrochemical deposition of p-NiTSPc was achieved by performing 50 cyclic scans in 2 mM NiTSPc aqueous solution prepared in NaOH 0.1 M. The obtained result is shown in Figure 2.

**Figure 3:**
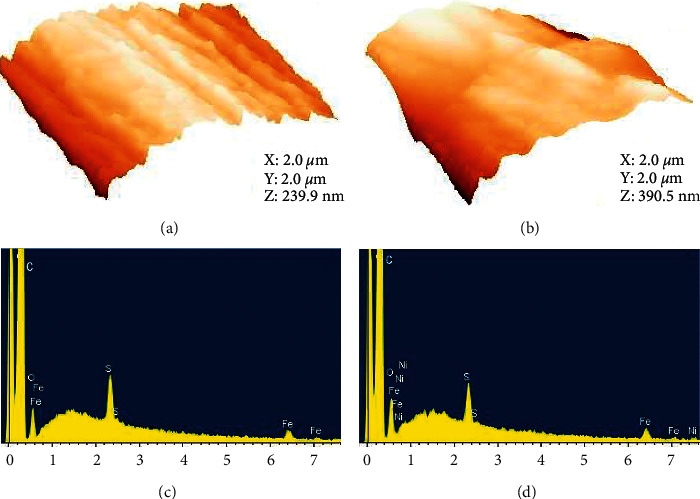
3D-AFM images of (a) unmodified CFME and (b) p-NiTSPc-CFME. EDX spectra of (c) unmodified CFME and (d) p-NiTSPc-CFME.

On Figure 2, redox signals were observed, respectively, at 0.35 and 0.45 V/SCE, traducing the electrodeposition of the p-NiTSPc film on the carbon fiber. The redox signals at 0.35 and 0.5 V/SCE obtained by cyclic voltammetry in NaOH 0.1 M with the new obtained modified electrode (inset of Figure 2) come to prove effectively the presence of the p-NiTSPc film on CFME. Moreover, in order to more appreciate the presence of the p-NiTSPc film on the carbon fiber, AFM images and EDX spectra were recorded for unmodified and modified CFME as shown in Figure 3.

As observed in Figures 3(a) and 3(b), a morphological difference was clearly seen between unmodified CFME and p-NiTSPc-CFME. In fact, the carbon fiber of the unmodified microelectrode (Figure 3(a)) shows a rough aspect, and this roughness is slightly weaker on the modified microelectrode (Figure 3(b)) due to the electrodeposition of the p-NiTSPc film. EDX spectra of unmodified CFME and p-NiTSPc-CFME (Figures 3(c) and 3(d), respectively) show the presence of common chemical elements such as carbon, oxygen, iron, and sulfur, characteristic of carbon fibers. Nickel atom is seen on the EDX spectrum of the modified microelectrode (Figure 3(d)), traducing the presence of the p-NiTSPc film on the carbon fiber. The obtained results in this section led us to evaluate the geometric and active areas of the elaborated microelectrodes.

### 3.2. Evaluation of Geometric and Active Areas of Both Working Electrodes

The geometric areas of both unmodified CFME and p-NiTSPc-CFME were evaluated using the formula Sg=*π𝔻ℓ*, where *π* = 3.14, *𝔻*=12 *μ*m, and *ℓ* = 0.6 cm. *𝔻* and *ℓ* are the diameter and length of the carbon fibers, respectively. Thus, both working microelectrodes (unmodified CFME and p-NiTSPc-CFME) have the same geometric surfaces, calculated to be 0.0022 cm^2^. The real surfaces of CFME and p-NiTSPc-CFME were estimated using the Randles Sevcik equation: Ip = K · n^3/2^ · A · D^1/2^ · C · V^1/2^ as recently reported by Mbokou et al. [16], where the constant K = 2.69 × 10^5^, n (= 2) is the number of electrons exchanged, A (cm^2^) is the real or active area of the electrode, D (= 0.62 × 10^−5^ cm^2^/s) is the diffusion coefficient, C (= 0.005 mol/L) is the concentration of the analyte, and V (= 0.1 V/s) is the scan rate. Thus, cyclic voltammograms were recorded on each working electrode in a 5 × 10^−3^ M [Fe(CN)_6_]^3−^ solution in 0.1 M PBS (pH 7.0) (see Figure S1, supplementary information). The obtained current intensities Ip1 = 0.35 *μ*A and Ip2 = 0.65 *μ*A for unmodified CFME and p-NiTSPc-CFME, respectively, helped to determine the real areas of 0.0015 cm^2^ and 0.0028 cm^2^ for unmodified CFME and p-NiTSPc-CFME, respectively. The obtained results show that the real area of p-NiTSPc-CFME is greater than that of unmodified CFME, due to the presence of p-NiTSPc film which increases the electrocatalytic properties of the modified electrode. The next section will be devoted to the electrochemical impedance spectroscopy that will confirm the electrocatalytic properties of the p-NiTSPc film.

### 3.3. Electrochemical Impedance Spectroscopy (EIS)

**Figure 4:**
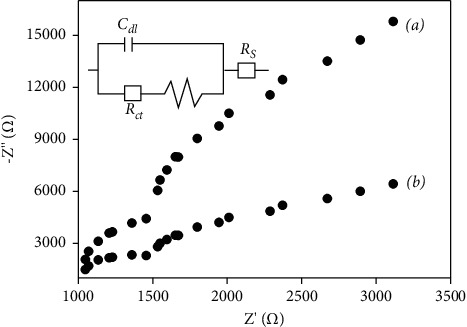
Nyquist plots of (a) CFME and (b) p-NiTSPc-CFME recorded in 0.1 M PBS (pH 7.0) containing 5 mM of [Fe(CN)_6_]^3−^. The frequency range was from 3 to 4 Hz at the formal potential of 0.30 V.

The electrical properties of the electrode/electrolyte system were studied using the EIS technique. Thus, the charge transfers of both tested electrodes were determined, and the obtained Nyquist diagrams are shown in Figure 4.

From Figure 4, the unmodified CFME showed an electron transfer resistance of 1000 Ω, while it was found to be about 500 Ω for p-NiTSPc-CFME. It is noticed that the electron transfer resistance decreases when modifying the electrode by the electrodeposition of p-NiTSPc-film. These results clearly confirm the electrocatalytic properties of the p-NiTSPc film and also prove its presence at the surface of the modified microelectrode.

### 3.4. Electrochemical Behaviour of Diuron at Different Electrodes

**Figure 5:**
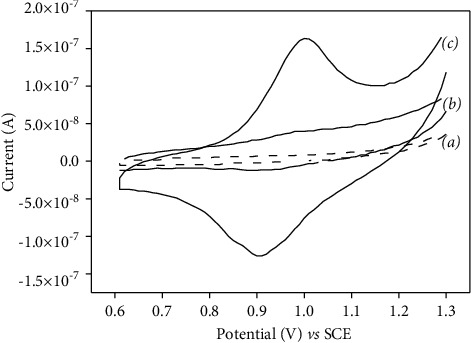
Cyclic voltammograms recorded in 0.1 M PBS containing 20 mg/L of diuron on (b) unmodified CFME and (c) p-NiTSPc-CFME. Curve (a) corresponds to the CV behaviour of p-NiTSPc-CFME in blank solution (baseline). Potential scan rate: 0.1 V/s.

Figure 5 presents the CV behaviour of both CFME and p-NiTSPc-CFME in the absence and presence of 20 mg/L diuron in 0.1 M PBS (pH 7.0).

**Scheme 1:**
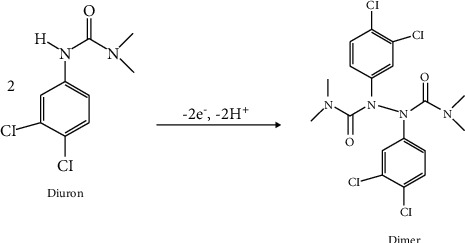
The electrochemical redox reaction mechanism of diuron.

From Figure 5 (a), no signal was observed on both tested microelectrodes in the blank PBS. In presence of diuron, a very low electrochemical response was obtained on CFME (Figure 5 (b)), indicating the poor electrocatalytic activity exhibited by the carbon fiber towards diuron oxidation. In comparison with NiTSPc-CFME (Figure 5 (c)), a couple of well-defined redox peaks was observed at the potentials of +0.9 V and +1 V/SCE, traducing the diuron redox reaction. This result is due to the presence of NiTSPc film on the carbon fiber which offers a large specific area and good conductivity to NiTSPc-CFME, leading to its enhanced electrocatalytic activity towards diuron. The electrochemical reaction of diuron leads to the formation of dimeric species as shown in Scheme 1 [8, 15]:

In fact, the formation of dimer involves two steps on NiTSPc-CFME. The first step corresponds to the oxidation reaction of the nickel(II) ion in the complex to (Ni^III^). The second step is the reaction occurring between the diuron and the oxidized complex, where there is a reduction of nickel and then an oxidation of diuron, forming the dimer [8, 13].

### 3.5. Effect of pH

**Figure 6:**
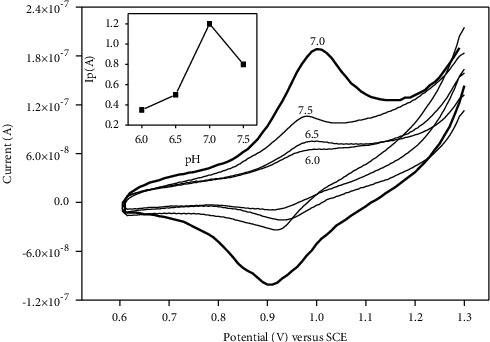
Cyclic voltammograms at different pH values in 20 mg/L diuron in 0.1 M PBS on p-NiTSPc-CFME. Insets show oxidation peak intensities against pH values.

The effect of PBS pH on the electrochemical response of 20 mg/L diuron at p-NiTSPc-CFME was investigated in the pH values ranging from 6.0 to 7.5 by cyclic voltammetry, and the obtained results are shown in Figure 6. As seen, no significant difference was observed on redox peak potential values at different pH values. The highest signal was obtained at the pH value of 7.0, traducing the easier migration of diuron at the surface of p-NiTSPc-CFME.

### 3.6. Effect of the Scan Rate

**Figure 7:**
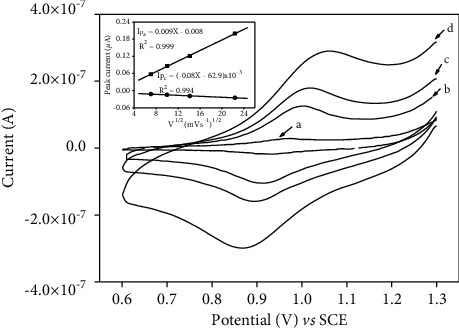
Cyclic voltammograms of 20 mg/L diuron in 0.1 M PBS (pH 7.0) on p-NiTSPc-CFME with different scan rates: (a) 0.01, (b) 0.05, (c) 0.1 and (d) 0.2 V/s. Inset plots of oxidation and reduction current versus square root scan rate.

Figure 7 shows cyclic voltammograms of 20 mg/L diuron in 0.1 M PBS (pH 7.0) on p-NiTSPc-CFME with different scan rates: 0.01, 0.05, 0.1, and 0.2 V/s. The obtained results (inset of Figure 7) show that the peak currents (anodic and cathodic) increase proportionally with the square root of the scan rate as expressed by the linear regression equations: Ipa (*μ*A) = 0.009 · *υ*^1/2^ (V^1/2^ · s^−1/2^) + 0.008 (R^2^ = 0.999) and Ipa (*μ*A) = −0.08 *υ*^1/2^ (V^1/2^ s^−1/2^) − 6.29 × 10^−3^ (R^2^ = 0.994). These results indicate that the redox reaction of diuron on p-NiTSPc-CFME is a diffusion-controlled process under the experimental conditions.

### 3.7. Optimization of SWV Parameters

The SWV technique was chosen and used in the present work for the quantitative analysis of diuron. In comparison to DPV (differential pulse voltammetry), SWV minimizes the contribution from the capacitive charging current to the current signal. This result was reported in our previous studies [16, 17]. Thus, SWV parameters such as frequency, amplitude, and step potential were initially varied. The optimized parameters for diuron determination were found to be frequency 50 Hz, amplitude 0.06 V, and step potential 0.02 V.

### 3.8. Calibration Curve

**Figure 8:**
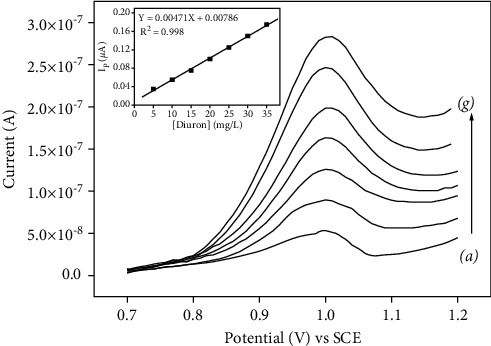
SWV of diuron at different concentrations in PBS on p-NiTSPc-CFME. Diuron concentrations (a–g) were 5, 10, 15, 20, 25, 30, and 35 mg/L. Insets show peak currents versus diuron concentration.

The relationship between the peak current and diuron concentrations was investigated by SWV on p-NiTSPc-CFME. Under optimal conditions, the oxidation peak current was proportional to the diuron concentration from 5 to 35 mg/L as shown in Figure 8. The obtained linear regression equation (inset of Figure 8) was Ipa (*μ*A) = 0.471 C (mg/L) + 0.786, with a correlation coefficient of R^2^ = 0.998. A detection limit (DL) was calculated to be 1.872 mg/L using the formula 3.3*σ*/m, where *σ* represents the standard deviation of the blank signal and *m* is the sensitivity. The limit of quantification (LQ), estimated using the formula LQ = 10*σ*/m was found to be 4.813 mg/L.

Table 1 presents the comparison of the obtained detection limit with others from the literature using different modifier electrodes for diuron determination. Despite the fact that the tested p-NiTSPc-CFME is a microelectrode, it exhibited comparable sensor performances with some macroelectrodes reported in the literature. As seen in Table 1, the obtained detection limit (8.030 *μ*M) was lower and even better [24] than that previously reported. Thus, the obtained results may be considered as a good starting point of reference for the electroanalysis of diuron using for the first time a carbon fiber microsensor.

### 3.9. Reproducibility and Stability

In order to evaluate the reproducibility and stability of the proposed electrode, p-NiTSPc-CFME was used for recording three times the square wave voltammograms (see Figure S2, supplementary information) in a solution containing 25 mg/L diuron in PBS (0.1 M, pH 7.0). The difference between the obtained signals was minor, and the relative standard deviation was calculated to be 4.9%.

### 3.10. Interference Study

In order to evaluate the selectivity of the proposed microsensor for the electrochemical determination of diuron, the effect of some inorganic ions such as Ca^2+^, Cl^−^, Na^+^, Al^3+^, Fe^2+^, Zn^2+^, K^+^, NH^4+^, Mg^2+^, NO_3_^−^, and SO_4_^2−^ was investigated under optimal conditions. Thus, known concentrations of each species were added to a solution containing 20 mg/L diuron in PBS (0.1 M, pH 7.0), and the SWV were recorded on p-NiTSPc-CFME. The tolerance limit of approximately ±5% was taken into consideration for each concentration of ions added. The obtained results (see Table S, supplementary information) showed that no significant interfering effect was observed on the diuron signal in presence of most of the listed elements, except both Fe^2+^ and Zn^2+^ ions, whose presence interferes on the oxidation signal of diuron. This result is in accordance with that previously published [15], where the presence of iron metal seriously affected the diuron signal by shifting its oxidation peak to positive values. In fact, the addition of Fe^2+^ in the solution containing diuron immediately changes the colouring and the pH value of the medium. These results were explained as a possible formation of a complex between the transition metal and the two molecules of diuron present in the medium [34].

### 3.11. Analytical Application of p-NiTSPc-CFME in Commercialized Agrochemicals

**Figure 9:**
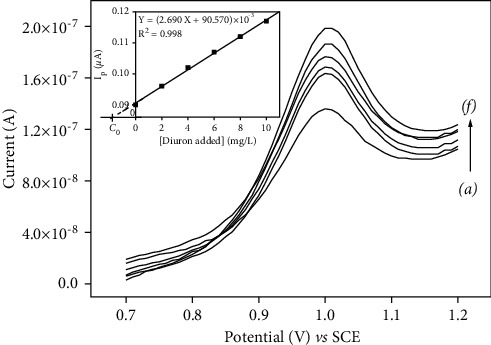
SWV curves recorded in 0.1 M PBS (pH 7.0) for the commercial diuron 80. Analytical conditions: [commercial diuron] = (a) unknown, (b–f) standard diuron added: 2, 4, 6, 8, and 10 mg/L. Inset displays the corresponding calibration curve.

The fabricated p-NiTSPc-CFME was successfully applied for quality control analysis in a commercial formulation of diuron. Thus, a solution of commercialized diuron 80 was prepared and diluted in 0.1 M PBS (pH 7.0). SWV on p-NiTSPc-CFME was used to determine the exact amount of diuron in the sample using the standard addition method.  Figure 9 shows the square wave voltammograms recorded in the sample (a) and at different concentrations of standard diuron added (b–f). The linear relationship obtained between the peak intensities (observed at 1 V/SCE) and the diuron concentrations (inset of Figure 9) was expressed by the equation Ip = (2.690 (diuron) + 90.570) × 10^−3^ (R^2^ = 0.998).

*C*_0_ represents the unknown concentration of diuron in the solution that was determined to be 1.83 mg/L by projection on the abscise axe as seen on the calibration curve (inset of Figure 9). The obtained results were summarised in Table 2, where the recovery amount of diuron in commercial diuron 80 was calculated to be 98.3%.

## 4. Conclusion

The present work shows for the first time the ability of a microelectrode in the electrochemical determination of diuron herbicide. In comparison to the unmodified CFME, p-NiTSPc-CFME exhibited a good electrocatalytic effect towards diuron detection. The calibration curve of p-NiTSPc-CFME was plotted in the concentration range from 21.450 to 150.150 *μ*M with a detection limit of 8.030 *μ*M (3.3 *σ*/m) and the limit of quantification of 20.647 *μ*M. The applicability of p-NiTSPc-CFME was addressed in a quality control analysis of an agrochemical formulation of diuron. The determination of diuron in the commercial formulation showed an acceptable recovery of 98.4%, suggesting that the proposed method can be suitable for the quality control analysis in agrochemical formulations.

## Figures and Tables

**Table 1:** Comparison of some methods reported for diuron determination.

Modified electrodes	pH	Linear range (*μ*M)	LD (*μ*M)	References
SPE-hapten/CNTs	8.0	0.0004 × 10^−4^–0.43	0.0004 × 10^−3^	[24]
^a^GCE-rGO-AuNPs/Nafion	2.0	0.001–0.01	0.0003	[25]
CPE/MWCNT-COOH^b^	8.0	0.05–1.25	0.009	[26]
SiO_2_@AuNPs/GCE	2.6	0.2–55	0.051	[27]
FeTAPc-SWCNT-GCE	4.0	50–100	0.260	[28]
NC-CPE	7.0	4.2–47	0.350	[15]
SPE/rGO/AuNPs^c^	5.5	2.15–128.7	0.536	[29]
MIP(DU)/MWCNTs-ISEs	2.5–4.5	3.2–1000	1.400	[30]
^d^GO-MWCNTs/GCE	5.0	9–380	1.490	[31]
CPE/NiOPc/hemin/GO^e^	8.0	9.9–150	6.140	[32]
CPE/hemin/Ni(II) complex	7.5	50–1000	20.000	[33]
p-NiTSPc-CFME	7.0	21.46–150	8.030	This work

^a^Reduced graphene oxide-gold nanoparticles/Nafion (rGO-AuNPs/Nafion). ^b^Carboxyl functionalized multiwalled carbon nanotubes (MWCNT-COOH). ^c^Reduced graphene oxide-gold nanoparticle (rGO-AuNP)-modified screen-printed electrode (SPE). ^d^Graphene oxide-multiwalled carbon nanotubes. ^e^Nickel(II) 1,4,8,11,15,18,22,25-octabutoxy-29H,31H-phthalocyanine complex.

**Table 2:** Electrochemical determination of diuron in commercial agrochemical formulation using p-NiTSPc-CFME (all values are in *g*, except the recovery in %).

Diuron trade name (*Diuron 80*)	Mass of diuron labelled on the sachet	250
	Theoretical mass of diuron in the sachet	200
	Experimental mass of diuron found using p-NiTSPc-CFME	197
	Recovery (%)	98.4

## Data Availability

The original data used in this study can be obtained from the submitting author if requested.

## Supplementary Materials

Supplementary Materials Figure S1: cyclic voltammograms recorded in 0.1 M PBS (pH 7.0) containing 5 mM of [Fe(CN)_6_]^3−^ on (a) unmodified CFME and (b) p-NiTSPc-CFME. Figure S2: square wave voltammograms recorded in 0.1 M PBS (pH 7.0) containing 25 mg/L diuron on p-NiTSPc-CFME. Table S: the effect of potential interfering species on the response of p-NiTsPc-CFME to 20 mg/L diuron in PBS.
