# Supplementary material for: Sensitive Carbon Fiber Microelectrode for the Quantification of Diuron in Quality Control of a Commercialized Formulation
Source: Int J Anal Chem. 2022 Mar 11;2022:9994639. doi: 10.1155/2022/9994639 (PMC8933123; doi:10.1155/2022/9994639)
Supplement: Supplementary Materials — Figure S1: cyclic voltammograms recorded in 0.1 M PBS (pH 7.0) containing 5 mM of [Fe(CN)6]3− on (a) unmodified CFME and (b) p-NiTSPc-CFME. Figure S2: square wave voltammograms recorded in 0.1 M PBS (pH 7.0) containing 25 mg/L diuron on p-NiTSPc-CFME. Table S: the effect of potential interfering species on the response of p-NiTsPc-CFME to 20 mg/L diuron in PBS. [file 9994639.f1.docx]

**Fig. S1** (Supplementary information): Cyclic voltammograms recorded in 0.1 M PBS (pH 7.0) containing 5 mM of [Fe(CN)_6_]^3-^ on (a) unmodified CFME and (b) p-NiTSPc-CFME. Potential scan rate: 0.1 V/s.

**Fig. S2** (Supplementary information): Square wave voltammograms recorded in 0.1 M PBS (pH 7.0) containing 25 mg/L diuron on p-NiTSPc-CFME.

**Table S** (Supplementary information): Eﬀect of potential interfering species on the response of p-NiTsPc-CFME to 20 mg/L Diuron in PBS.

| Interfering ions | Concentration (mg/L) of added ions | % variation of the peak current in SWV (with I_diuron_ = 100%) |
| --- | --- | --- |
| Ca^2+^ | 2 | 0 |
|  | 4 | 0 |
| Cl^-^ | 2 | 0 |
|  | 4 | 0 |
| Na^+^ | 2 | 0 |
|  | 4 | 0 |
| K^+^ | 2 | 0 |
|  | 4 | 0 |
| NH_4_^+^ | 2 | 0 |
|  | 4 | 0 |
| Al^3+^ | 2 | 0 |
|  | 4 | 0 |
| Mg^2+^ | 2 | 0 |
|  | 4 | 0 |
| SO_4_^2-^ | 2 | 0 |
|  | 4 | 0 |
| NO_3_^-^ | 2 | 0 |
|  | 4 | 0 |
| Zn^2+^ | 2 | -5 |
|  | 4 | -12 |
| Fe^2+^ | 2 | -15 |
|  | 4 | -35 |
